# Round about the Asylums.—VI

**Published:** 1888-05-12

**Authors:** 


### Round About the Asylums.—VI.

By a Medical Superintendent.

### HULL BOROUGH ASYLUM.

On the last day of the year the number of patients was 254, being oddly enough the same as on the last day of the previous year. There were 84 admissions, 41 discharges, and 43 deaths; and these yield the following percentages: Recoveries, 28·39 per cent. on the admissions; deaths, 16·66 per cent. on the average number resident. The admissions seem to have been very unfavourable. At least twenty were afflicted with general paralysis, and many others must have been sent to the asylum in a hopeless state, as fourteen died within three months of admission. During the summer the water supply failed totally for a few days, and water had to be carted from Willerby for the use of the asylum; a state of things so serious, that the visitors wisely decided to lay on the town water, and this was not done too soon, for the supply failed again during the winter months. The weekly cost of maintenance is 10*s.* 3*d.* Private patients are received, and wards have been specially furnished to meet their wants; but it would seem that as yet few have taken advantage of the privilege. Employment of the patients is carefully attended to, and the high proportion of 173 out of 254 are occupied in one way or other. The Commissioners in Lunacy say that the case books are well kept, and that the general state of the asylum is creditable to Dr. Merson. The asylum is almost new, and yet we read that the woodwork and plaster are giving way in many places, and the Commissioners add that they "cannot congratulate the committee upon the way the work has been executed." Does this refer to all the work or only to the wood and plaster, and what has the architect to say in answer to the Commissioners' strictures?

### OXFORD COUNTY ASYLUM.

The numbers at the close of the year were 216 men and 277 women. Eighty-five patients were admitted during the year, 38 were discharged, and 51 died. The recoveries were 26 per cent. on the admissions, and the deaths 10 per cent. on the average number resident. The weekly cost was a fraction under 8*s.* 5*d.* The Commissioners remark on the number of minor accidents, and they think that these may be owing to an insufficient staff. They add, however, that the patients were very quiet and orderly on the day of their visit. Two hundred and sixty-four of the patients are employed; 52 are in the garden, over 100 are in the wards and offices, 51 at needlework, and but very few in the workshops.

### NOTTINGHAM COUNTY ASYLUM.

In former days this was known as the General Lunatic Asylum for Nottingham. It used to contain both private and borough patients, as well as its own county cases; but the Coppice now takes most of the private patients, and the Borough Asylum has sprung up, mushroom-like, and now overshadows the parent institution. This, its seventy-seventh annual report, shows that it contains only 312 patients at present, and 47 of these do not belong to the county. The recoveries stand at 34·66 per cent. on the admissions, and the deaths are 15 per cent. on the average number resident There was no epidemic to account for this high mortality, and the average age at death was 51 for the men, and 55 for the women. There was one suicide, the first for fifteen years, and the asylum was twice on fire, but the damage done was slight on both occasions. The weekly cost of maintenance was 9*s*. 5*d*., but owing to the existence of a reserve fund, it was possible to charge only 8*s*. 6*d*. to the unions, a proceeding more in harmony with the ratepayers' feelings than with the Lunacy Acts. Being in the town of Nottingham, the site of this asylum is much cramped, and the Commissioners hope that the question of removing it into the country may not be lost sight of. We congratulate Dr. Aplin on his recovery from a severe attack of rheumatic fever.

### CITY OF LONDON ASYLUM.

During the year this asylum lost the services of the medical superintendent, Dr. Jephson, who, after twenty-three years' service retired on the handsome pension of £800 a-year. Dr. Ernest White, formerly assistant medical officer at the Kent County Asylum, now holds the reins of office. The changes among the staff seem to have been extremely numerous. The assistant medical officer, the housekeeper, and the head nurse are all new officers, and ten of the nurses have had under one year's service. Dr. White must have had his hands full for awhile; but to begin new duties with a new staff is not always a thing to be regretted. There were 421 patients resident at the end of the year. The recoveries were 31·92 on the admissions, and the deaths were five per cent. on the average number resident. A course of lectures on nursing was given by the assistant medical officer, and the visitors granted some money for distribution of prizes among the class. This is a step in the right direction. The delivery of these lectures and the rewards are alike deserving of much praise, and we hope Dr. Greenlees will persevere in his good work, and that the visitors will repeat their grant. There were six escapes, but all, save one, were captured, and brought back to the asylum. The building is about to be added to, and the additions will accommodate 128 patients.

